# Erratum for Franco et al., “A Novel Secreted Protein, MYR1, Is Central to *Toxoplasma*’s Manipulation of Host Cells”

**DOI:** 10.1128/mBio.01869-18

**Published:** 2018-10-02

**Authors:** Magdalena Franco, Michael W. Panas, Nicole D. Marino, Mei-Chong Wendy Lee, Kerry R. Buchholz, Felice D. Kelly, Jeffrey J. Bednarski, Barry P. Sleckman, Nader Pourmand, John C. Boothroyd

**Affiliations:** a Department of Microbiology and Immunology, Stanford University School of Medicine, Stanford, California, USA; b Biosciences and Biotechnology Division, Lawrence Livermore National Laboratory, Livermore, California, USA; c Department of Biomolecular Engineering, University of California, Santa Cruz, California, USA; d Department of Pediatrics, Washington University in St. Louis, St. Louis, Missouri, USA; e Department of Pathology and Immunology, Washington University in St. Louis, St. Louis, Missouri, USA

## ERRATUM

Volume 7, no. 1, e02231-15, 2016, https://doi:10.1128/mBio.02231-15. Upon reexamining the original sequencing data for MFM2.1, we have determined that Table 1 has an error. While 2% of reads did show the indicated tCa-to-tTa mutation at nucleotide 3288737 in the ROP17 gene, 42% of reads indicated a nonsense mutation (tCa to tGa) at this position, making the change in the amino acid sequence S151*. This does not change any of the biological conclusions of the paper.

